# Fertilizer type and humic acid improve the growth responses, nutrient uptake, and essential oil content on *Coriandrum sativum* L.

**DOI:** 10.1038/s41598-022-11555-4

**Published:** 2022-05-06

**Authors:** Farzad Rasouli, Yousef Nasiri, Mohammad Asadi, Mohammad Bagher Hassanpouraghdam, Sina Golestaneh, Yaghoub Pirsarandib

**Affiliations:** 1 Department of Horticultural Science, Faculty of Agriculture, University of Maragheh, Maragheh, Iran; 2 Department of Plant Production and Genetics, Faculty of Agriculture, University of Maragheh, Maragheh, Iran

**Keywords:** Plant sciences, Plant ecology, Secondary metabolism

## Abstract

In recent decades, the over-use of chemical fertilizers has imposed many environmental challenges worldwide. Nowadays, organic fertilizers such as vermicompost and livestock manure have gained a huge interest in sustainable agricultural systems. A 2-year field research was conducted as factorial based on a randomized complete block design to assay the fertilizer and humic acid (HA) efficiency on the growth responses and essential oil composition of *Coriandrum sativum*. The treatments were different fertilizer sources (livestock manure, vermicompost, and chemical fertilizers) and humic acid fertigation before and at the beginning of the flowering stage. The highest protein content was observed under vermicompost × HA application before flowering (0.118 μmol L^−1^ and 0.128 μmol L^−1^, respectively). Moreover, the co-application of organic fertilizers × HA at the beginning of flowering resulted in a significant increase in the photosynthetic pigments and N, P, K, Fe, Zn, and Mn content. According to the GC-FID and GC–MS analysis, linalool (55.91–63.19%), γ-terpinene (4.65–6.13%), *α*-pinene (2.64–5.74%), geranyl acetate (3.49–5.51%), 2-dodecanal (2.92–4.46%), menthol (1.33–3.90%), *p*-cymene (1.73–2.24%), and geraniol (1.25–2.15%) were the main essential oil constituents. The top linalool content was obtained by using chemical fertilizers and vermicompost × HA at the flowering onset stage. In general, the results revealed that chemical fertilizers could be replaced with vermicompost × HA and their co-application positively influenced the growth responses and the essential oil composition of coriander. Furthermore, the results obtained would be advisable to the extension section and the pioneer farmers to amend the large-scale production systems in favor of environmental health.

## Introduction

Climatic conditions, soil factors, and mineral nutrients are fundamental in the production of agricultural crops. In recent decades, the excessive use of chemical fertilizers in conventional agriculture has caused many environmental problems. These include the pollution of soil and water resources, reduced quality of food products, and disturbance of soil biological balance, which cause irreplaceable damage to the ecosystem^1^. The global approach to establishing a sustainable agricultural system has changed with the use of new management methods, which necessitates paying attention to the biological and integrated systems, particularly organic fertilizers, to partially fulfill the fertilizer needs of plants and reduce the use of chemical fertilizers^2^. One of the basic principles in sustainable agriculture is using organic fertilizers, such as vermicompost, humic acid, and livestock manure. These are cost-effective, economically acceptable, and environmentally friendly organic fertilizers and a rich source of macro-and micronutrients, vitamins, enzymes, and growth-promoting hormones, which play an essential role in sustaining soil fertility and increasing the yield and quality of crops and medicinal plants^3^.

Vermicompost is a rich organic fertilizer with adequate levels of humic substances and nutrients available for the plants. It is produced by the activity of various species of earthworm species such as *Eisenia fetida*, *Eisenia hortensis*, and other earthworms^4^. Organic acids produced during the vermicompost processing absorb micronutrients, such as Fe, Zn, Cu, etc., and gradually make them available to plants^5^. Due to its high-water holding capacity, vermicompost always provides adequate amounts of water that prevent severe water deficit stress in plants as well^6^.

Humic acid (HA) is a natural organic polymeric compound produced from soil organic matter, peat, and lignin decay. It absorbs several ions to form chelates with micronutrients, which release the ions slowly and continuously^7^. HA contains many carboxyls, phenolic, carbonyl, and hydroxyl chemical groups attached to aliphatic or aromatic carbons and improves the plants’ stress tolerance, growth potential, seed germination speed and rate, crop quality, and yield, as well as the soils fertility and physicochemical properties such as permeability, aeration, granulation, and soil water holding capacity^8^.

Coriander (*Coriandrum sativum* L.) is an aromatic, annual medicinal herb of the Apiaceae family. Coriander is originated from the Mediterranean region, Northern Africa, and Southwest of Asia. The seeds’ essential oil is considered to have antioxidant and antimicrobial properties in spoilage prevention and food preservation^9^. The most important constituents of coriander essential oil are linalool, citronellol, caryophyllene oxide, *cis*-4-decal propylene lactone, and caprolactone, which have anti-inflammatory analgesic, anticonvulsant, blood pressure-lowering, and cholesterol-lowering properties. The essential oil is also used in food preparation, perfumery, cosmetics, and medicine^10,11^.

Several studies are revealing that the application of organic fertilizers and bio-stimulants improve plant height, fresh and dry matter yield, micronutrients uptake, the number of branches, and essential oil content (EOC) in medicinal plants such as *Mentha piperita* L.^12^, *Salvia officinalis*^13^, *Mentha arvensis* L.^14^, and *Carum carvi* L.^15^. Hassan and Fahmy reported that the foliar application of HA significantly increased the yield components and EOC in chamomile^16^. Dehsheikh et al.^17^ found that HA application increased soil organic matter content and improved basil plants' growth potential and essential oil yield (EOY).

The main idea in the production of medicinal plants is the organic production of high-quality crops. Hence, the use of organic fertilizers and bio-stimulants has become more important to fulfill the nutritional needs of farmlands. Considering, this study aimed (i) to evaluate and compare the HA fertigation as a growth stimulant under the organic and chemical fertilizers regime in coriander, (ii) to compare the HA fertigation time (before flowering and after full bloom), and (iii) to assess the growth responses and essential oil components under organic and chemical fertilizers use.

## Results and discussion

### Plant height

**Figure 1:**
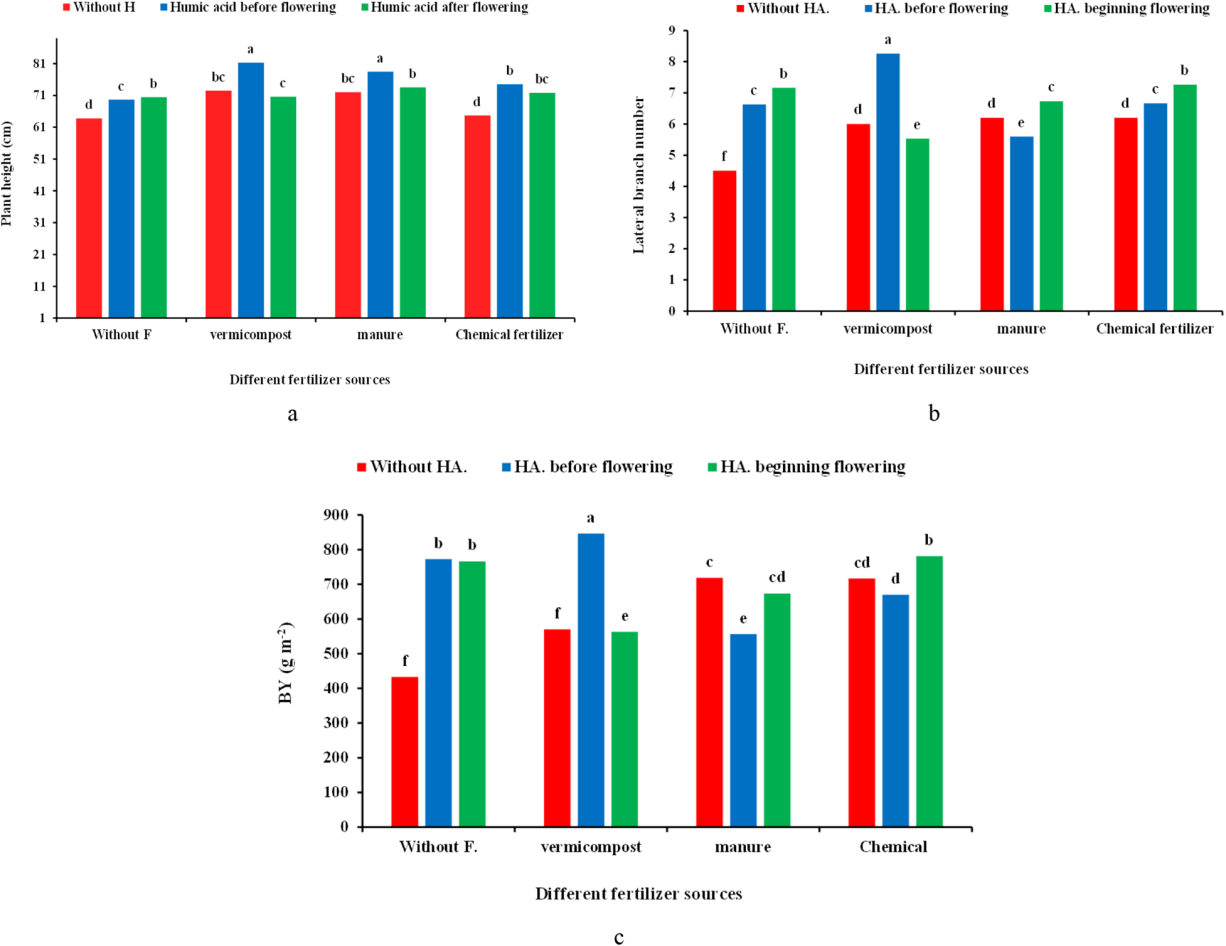
Effect of different fertilizer sources × humic acid (HA) treatments on the plant height (**a**), the lateral stem number (**b**), and biological yield (BY) (**c**) of coriander. Different letters indicate significant differences according to LSD test P < 0.05 (average of two years).

The results showed that plant height was affected by HA application and the interaction of the fertilizers × HA. Still, this trait was not influenced by the sole effect of the fertilization source (Table 1). The top plant height was obtained in vermicompost and livestock manure × HA application before flowering (81.27 cm and 78.47, respectively). In comparison, the lowest was recorded in control (63.77 cm), which was noted 27.42 and 23.05% increment compared to the control, respectively (Fig. 1a).

### Lateral branch number

The lateral stems number was significantly influenced by different levels of fertilizers × HA fertigation (Table 1). The application of vermicompost × HA before flowering resulted in the highest number of lateral stems (8.27), which was 83.77% more than control (Fig. 1b).

### Biological yield (BY)

The BY of coriander was significantly affected by the various fertilizers source × HA (Table 1). The uppermost BY was noted (846.7 g m^−2^) in vermicompost × HA application before flowering, showing an increase of 95.54% compared to the control (433 g m^−2^) (Fig. 3c).

### Plant dry weight (DW)

**Figure 2:**
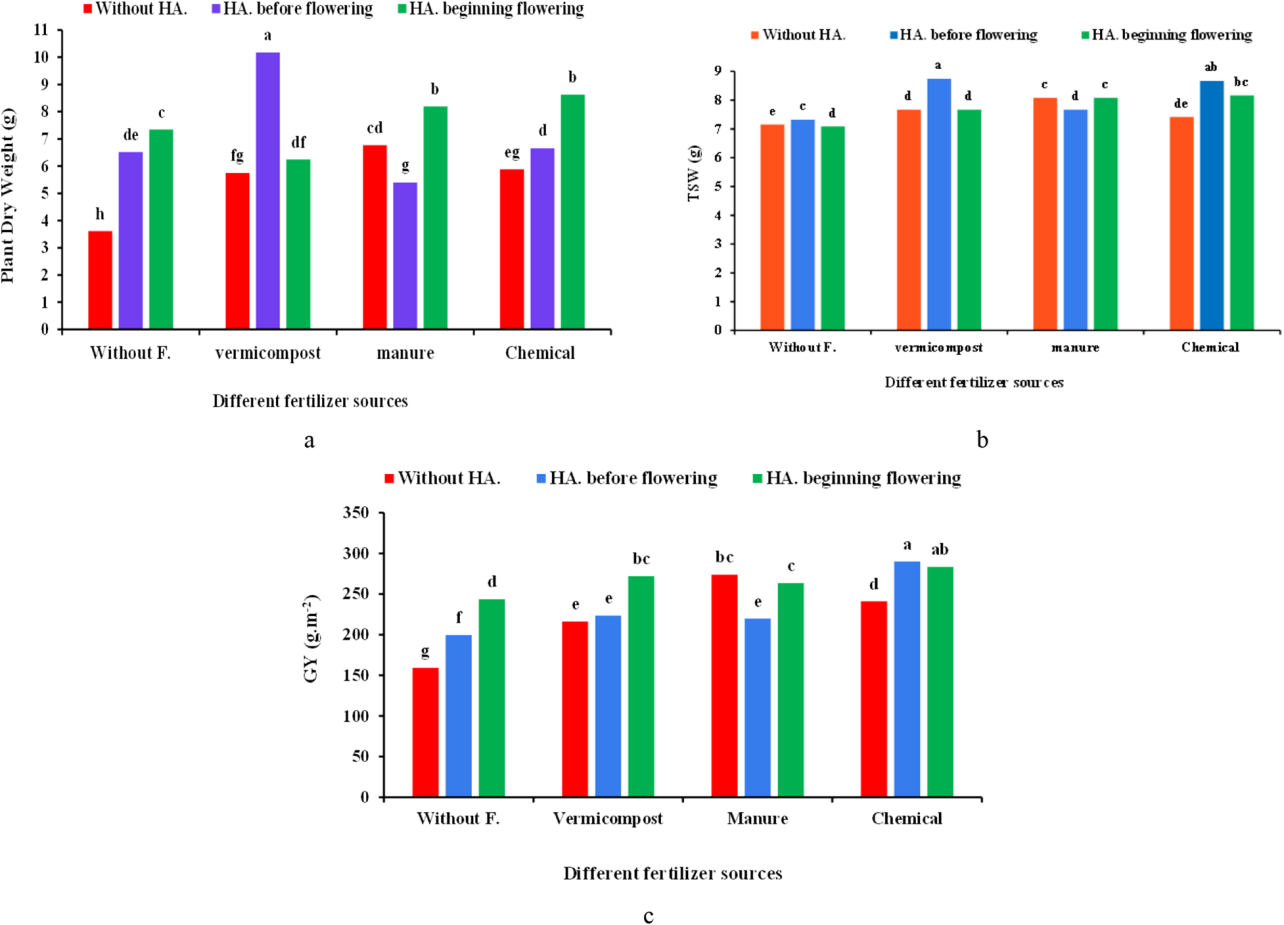
Effect of different fertilizers sources × humic acid (HA) treatments on the plant dry matter (**a**), Thousand-seeds weight (TSW) (**b**), and grain yield (GY) (**c**) of coriander. Different letters indicate significant differences according to LSD test P < 0.05 (average of two years).

The different fertilizer source and HA, and their combination significantly affected plant DW (Table 1). The highest plant DW was observed by applying vermicompost × HA fertigation before flowering (10.18 g), which was up to 182% more than control (3.61 g) (Fig. 2a).

### Thousand seeds weight (TSW)

The effects of different fertilizers source and HA and their interaction effects were significant on the coriander TSW (Table 1). The application of vermicompost × HA fertigation before flowering led to the highest TSW (8.750 g), that was 22.37% higher than control (7.15 g) (Fig. 2b).

### Grain yield (GY)

Various fertilizers source × HA significantly influenced GY (Table 1). The highest GY (290.2 g m^−2^) was achieved by chemical fertilizer × HA application at the beginning of flowering, but it was not significantly different from vermicompost × HA at the same stage. The least GY (152.2 g m^−2^) belonged to the control, 90.67% lower than the superior treatment (Fig. 2c).

### Photosynthetic pigments content

**Figure 3:**
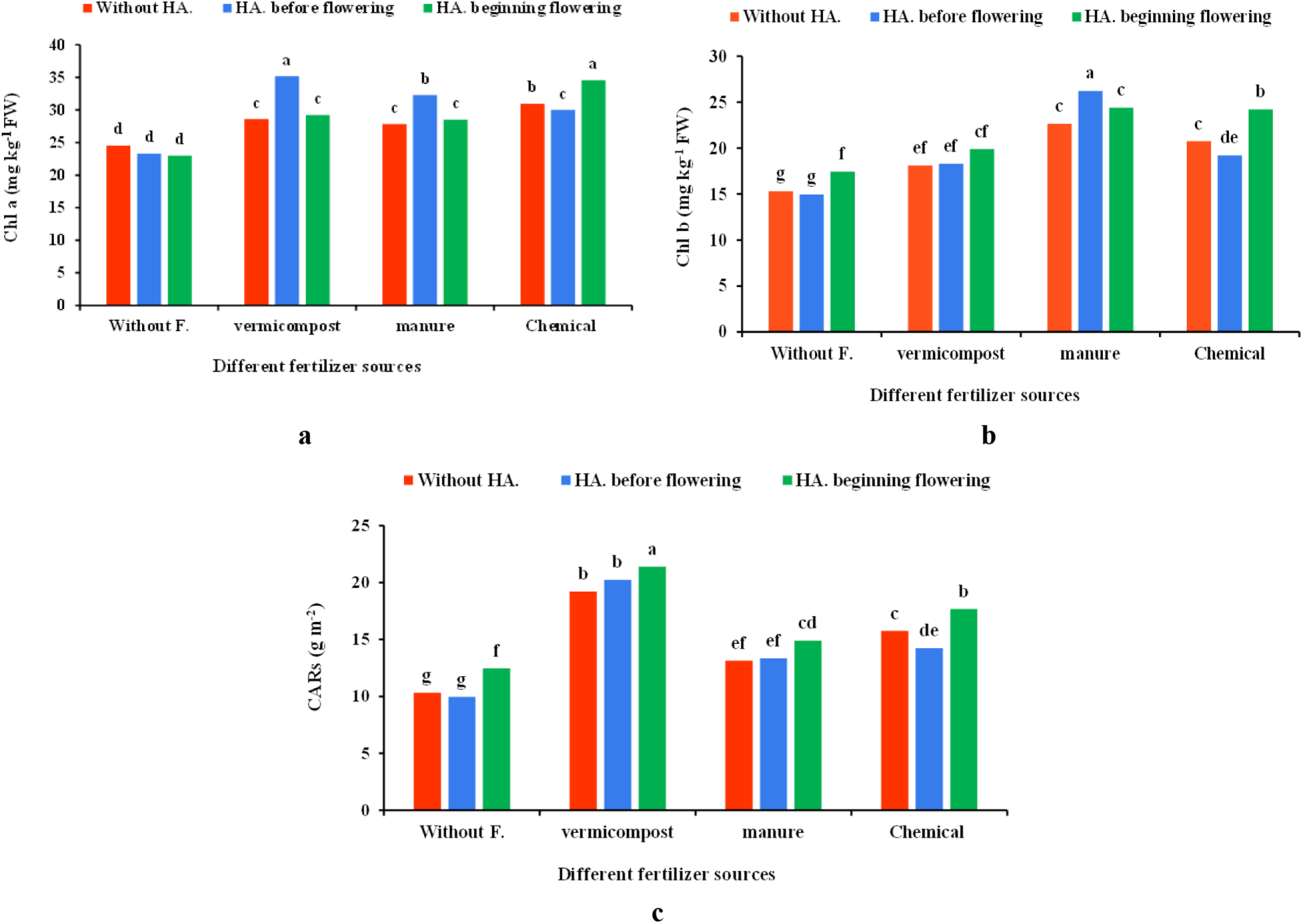
Effect of different fertilizer sources × humic acid (HA) treatments on chlorophyll a (Chl *a*) (**a**), chlorophyll b (Chl *b*) (**b**), and carotenoids (CARs) (**c**) of the coriander plant. Different letters indicate significant differences according to LSD test P < 0.05 (average of two years).

The results revealed that the various fertilizers source × HA significantly improved chlorophyll a (Chl *a*) content (Table 1). The highest content of Chl *a* was obtained in the vermicompost × HA before flowering (35.17 mg kg^−1^ FW), and in the application of chemical fertilizer × HA at the beginning of flowering (34.56 mg kg^−1^ FW), which were 43.31% and 40.83% higher than control, respectively (Fig. 3a). The lowest Chl *a* content was recorded in control (24.54 mg kg^−1^ FW), which had a non-significant difference with the application of HA before (23.3 mg kg^−1^ FW) and at the beginning (22.98 mg kg^−1^ FW) of flowering.

The findings showed that the different fertilizers source, HA, and their interaction significantly affected coriander plants' chlorophyll b (Chl b) content (Table 1). The highest Chl *b* content (26.24 mg kg^−1^ FW) was obtained in livestock manure × HA before flowering, which showed an increase of up to 61.71% compared to the control (15.32 mg kg^−1^ FW). The lowest Chl *b* content was recorded in control and HA treatments before flowering (Fig. 3b).

**Figure 4:**
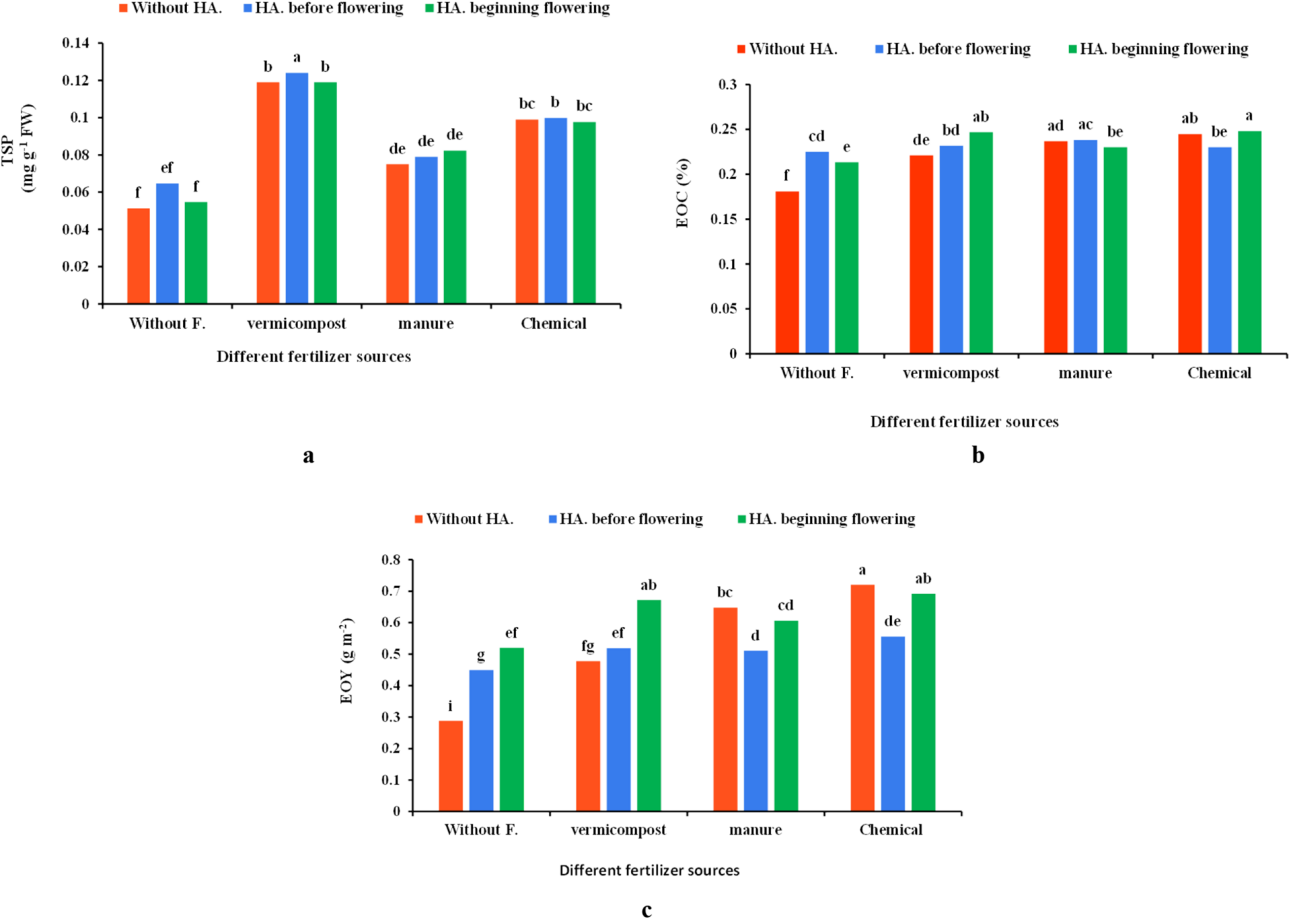
Effect of different fertilizer sources × humic acid (HA) treatments on TSP (**a**), essential oil content (EOC) (**b**), essential oil yield (EOY) (**c**), and total soluble protein content (TSP) (**d**) of coriander plants. Different letters indicate significant differences according to LSD test P < 0.05 (average of two years).

Moreover, the effects of various fertilizers sources, HA, and their co-application were significant on the carotenoids (CARs) content (Table 1). The highest CARs content (20.8 g m^−2^) was observed in the application of vermicompost × HA at the beginning of flowering, which was 101.55% higher than control. The lowest content of CARs was traced in control (10.32 g m^−2^) and HA (9.95 g m^−2^) before the flowering stage (Fig. 4c).

### Total soluble proteins (TSP) content

The results revealed that different fertilizers and their interaction with HA significantly increased TSP, but HA did not affect the trait (Table 1). The highest TSP was achieved with vermicompost × HA before flowering (0.128 mg g^−1^ FW), which was 149.51% more than control. The least TSP content was detected in control (0.0513 mg g^−1^ FW) and HA (0.0646 mg g^−1^ FW) before flowering and at the beginning (0.546 mg g^−1^ FW) of flowering (Fig. 4a).

### Essential oil content (EOC)

Various fertilizers source, HA, and interactions significantly influenced EOC (Table 1). Different fertilizers combined with HA fertigation increased EOC up to 30–37% compared to control. The highest EOC was obtained with chemical fertilizer × HA at the beginning of flowering (0.248%), chemical fertilizer × without HA (0.244%), vermicompost × HA at the beginning of flowering (0.246%), manure × HA before flowering (0.238%) and manure × without HA (0.236%). The least EOC was recorded in the control (0.181%) (Fig. 4b).

### Essential oil yield (EOY)

The effects of different fertilizers type, HA, and their combinations were significant on the EOY (Table 1). Chemical fertilizer × without HA, chemical fertilizer × HA at the beginning of flowering and vermicompost × HA at the beginning of flowering led to the highest EOY (0.720 g m^−2^, 0.692 g m^−2^ and 0.671 g m^−2^, respectively). While, the least EOY was recorded in control with 0.288 g m^−2^ (Fig. 4c). The co-application of various fertilizers and HA improved EOY up to 132–150% over the control.

### Essential oil constituents

Twenty seven constituents were identified, accounting for 90–97% of the total essential oil. Linalool (63.99%) was the predominant constituents of coriander seed EO. In addition, γ-terpinene (6.13%), α-pinene (5.74%), geranyl acetate (5.51%), dodecanal (4.46%), menthol (3.90%), *p*-cymene (2.24%), and geraniol (2.15%) were identified as the other predominant constituents (Table 2). The highest hydrocarbons and oxygenated monoterpenes were observed in the chemical fertilizer × HA application before and at the beginning of flowering, respectively (Table 3). At the beginning of flowering, the highest content of sesquiterpene hydrocarbons was recorded in the no-fertilizer × HA treatment (Table 4).

**Table 1:** Variance analysis for the growth traits, minerals content and essential oil content and yield of coriander plants in response to the chemical and organic fertilizers as well as humic acid application.

S.O.V	df	Mean square											
		Plant height (cm)	Lateral branch number	BY (g m^−2^)	Plant dry weight (g)	TSW (g)	GY (g m^−2^)	Chl *a* (mg kg^−1^ FW)	Chl *b* (mg kg^−1^ FW)	CARs (mg kg^−1^ FW)	TSP (mg g^−1^ FW)	EOC	EOY (g m^−2^)
R	2	10.966*	0.060*	93.083*	0.515*	0.064*	224.06**	0.067ns	4.120**	4.126*	0.0001**	0.0001*	0.005**
F	3	0.9025ns	2.543**	63,034.8**	10.289**	0.479**	6450.7**	22.87**	35.80**	35.78**	0.004**	0.001**	0.064**
HA	2	182.35**	11.674**	133,468.0**	26.080**	2.044**	3949.1**	1.468ns	4.872**	4.875**	0.007ns	0.0001**	0.022**
F × HA	6	72.308**	0.379 **	3099.19**	1.884**	0.148**	6327.1**	86.77**	51.71**	51.69**	0.003**	0.001**	0.054**
Error	22	2.891	0.052	791.26	0.155	0.032	70.707	1.371	0.563**	0.564	0.001	0.0001	0.001
C.V		7.36	10.61	9.18	10.81	7.25	8.56	8.98	9.87	12.43	10.23	8.81	10.11

*, ** and ns, significant at the 5% and 1% probability levels and non-significant, respectively.

**Table 2:** Composition of the coriander essential oil influenced by the organic and chemical fertilizers × humic acid application (average of 2 years).

No	Treatments														
	Components	RI LIT. RI	RI LIT. RI	T1	T2	T3	T4	T5	T6	T7	T8	T9	T10	T11	T12
1	n-Nonane	897.50	**900**	0.08 ± 0.03	0.15 ± 0.02	0.16 ± 0.02	0.12 ± 0.00	0.13 ± 0.01	0.018 ± 0.00	0.09 ± 0.02	0.13 ± 0.00	0.10 ± 0.02	0.21 ± 0.07	0.16 ± 0.00	0.18 ± 0.01
2	*α*-Pinene	927.83	**932**	**2.46 ± 1.43**	5.69 ± 0.04	4.35 ± 0.18	4.42 ± 0.32	4.38 ± 0.45	5.52 ± 0.16	4.90 ± 0.67	5.21 ± 0.14	5.50 ± 0.18	5.17 ± 0.32	4.81 ± 0.44	**5.74 ± 0.90**
3	5-Methylnonane	953.07	**958**	0.4 ± 0.00	0.25 ± 0.06	0.54 ± 0.07	0.34 ± 0.01	0.44 ± 0.04	0.38 ± 0.00	0.39 ± 0.02	0.33 ± 0.02	0.40 ± 0.00	0.41 ± 0.07	0.47 ± 0.09	0.40 ± 0.00
4	3-Mrthylnonane	964.36	**970**	0.45 ± 0.03	0.35 ± 0.01	0.49 ± 0.07	0.35 ± 0.01	0.38 ± 0.01	0.35 ± 0.03	0.34 ± 0.01	0.37 ± 0.01	0.38 ± 0.00	0.32 ± 0.00	0.33 ± 0.01	0.50 ± 0.05
5	Sabinene	966.80	**969**	0.85 ± 0.06	0.77 ± 0.03	0.61 ± 0.11	0.70 ± 0.01	0.61 ± 0.04	0.74 ± 0.02	0.73 ± 0.01	0.48 ± 0.10	0.77 ± 0.02	0.57 ± 0.09	0.75 ± 0.09	0.62 ± 0.11
6	Pinene < β>	969.56	**974**	0.72 ± 0.08	0.82 ± 0.00	0.76 ± 0.02	0.78 ± 0.02	0.79 ± 0.09	0.82 ± 0.06	0.58 ± 0.14	0.78 ± 0.02	0.71 ± 0.10	0.64 ± 0.00	0.74 ± 0.01	0.78 ± 0.06
7	beta Myrcene	986.50	**988**	0.90 ± 0.04	0.83 ± 0.09	0.91 ± 0.02	0.78 ± 0.00	0.72 ± 0.04	0.79 ± 0.02	0.78 ± 0.02	0.80 ± 0.00	0.78 ± 0.07	0.79 ± 0.01	0.73 ± 0.06	0.97 ± 0.01
8	p-Cymene	1018.90	**1020**	**2.24 ± 0.13**	1.87 ± 0.19	2.09 ± 0.05	1.79 ± 0.06	2.12 ± 0.02	2.04 ± 0.14	2.23 ± 0.22	1.91 ± 0.11	2.15 ± 0.14	1.74 ± 0.24	**1.73 ± 0.33**	2.090 ± 0.11
9	Cineole <1 ,8−>	1024.68	**1026**	0.33 ± 0.06	0.27 ± 0.01	0.45 ± 0.10	0.45 ± 0.10	0.38 ± 0.08	0.21 ± 0.01	0.31 ± 0.04	0.29 ± 0.04	0.27 ± 0.02	0.33 ± 0.03	0.42 ± 0.10	0.51 ± 0.11
10	γ-Terpinene	1053.21	**1059**	5.25 ± 0.16	5.54 ± 0.35	5.79 ± 0.37	4.82 ± 0.38	4.72 ± 0.06	6.05 ± 0.36	5.32 ± 0.34	**6.13 ± 0.52**	**4.65 ± 0.02**	5.67 ± 0.44	6.00 ± 0.06	5.89 ± 0.92
11	*cis*-Sabinene hydrate	1060.45	**1065**	0.183 ± 0.01	0.18 ± 0.01	0.27 ± 0.05	0.17 ± 0.03	0.22 ± 0.05	0.24 ± 0.00	0.27 ± 0.03	0.26 ± 0.05	0.18 ± 0.02	0.22 ± 0.02	0.23 ± 0.02	0.16 ± 0.01
12	*n*-Octanol	1067.49	**1071**	0.27 ± 0.06	0.26 ± 0.05	0.28 ± 0.03	0.31 ± 0.07	0.25 ± 0.04	0.36 ± 0.07	0.33 ± 0.06	0.16 ± 0.01	0.18 ± 0.01	0.20 ± 0.00	0.25 ± 0.02	0.28 ± 0.02
13	Linalool	1101.23	**1095**	**55.91 ± 0.55**	62.66 ± 1.57	59.38 ± 2.35	56.92 ± 3.16	62.24 ± 0.44	62.11 ± 0.97	60.19 ± 0.90	59.94 ± 2.43	**63.19 ± 0.19**	62.63 ± 1.17	61.18 ± 0.23	59.06 ± 0.17
14	Camphor	1136.31	**1141**	0.26 ± 0.03	0.27 ± 0.02	0.35 ± 0.08	0.53 ± 0.14	0.47 ± 0.16	0.54 ± 0.00	0.34 ± 0.10	0.30 ± 0.09	0.27 ± 0.04	0.36 ± 0.03	0.31 ± 0.06	0.28 ± 0.03
15	Citronellal	1148.59	**1148**	0.33 ± 0.03	0.28 ± 0.04	0.22 ± 0.01	0.26 ± 0.00	0.20 ± 0.04	0.30 ± 0.00	0.21 ± 0.04	0.17 ± 0.03	0.26 ± 0.01	0.24 ± 0.01	0.27 ± 0.01	0.21 ± 0.01
16	Menthofuran	1158.12	**1159**	0.44 ± 0.03	0.36 ± 0.02	0.46 ± 0.06	0.71 ± 0.15	0.40 ± 0.06	0.42 ± 0.02	0.30 ± 0.03	0.57 ± 0.17	0.29 ± 0.02	0.31 ± 0.02	0.33 ± 0.04	0.4 ± 0.07
17	Menthol	1166.93	**1167**	**3.90 ± 1.14**	1.65 ± 0.42	2.04 ± 0.15	2.43 ± 0.03	1.86 ± 0.57	**1.33 ± 0.23**	1.34 ± 0.18	3.24 ± 0.74	1.75 ± 0.35	2.61 ± 0.21	3.44 ± 0.89	1.75 ± 0.29
18	Terpinen-4-ol	1170.50	**1174**	0.42 ± 0.05	0.33 ± 0.06	0.45 ± 0.10	0.27 ± 0.1	0.27 ± 0.01	0.32 ± 0.02	0.27 ± 0.01	0.28 ± 0.01	0.27 ± 0.01	0.24 ± 0.00	0.29 ± 0.02	0.29 ± 0.03
19	*a*-Terpineol	1183.94	**1186**	1.45 ± 0.15	**1.12 ± 0.01**	1.75 ± 0.22	1.23 ± 0.01	1.28 ± 0.02	1.30 ± 0.05	1.26 ± 0.02	1.25 ± 0.06	1.24 ± 0.04	1.36 ± 0.12	1.45 ± 0.5	**1.56 ± 0.03**
20	Dodecane <n−>	1196.04	**1200**	0.33 ± 0.04	0.24 ± 0.00	0.34 ± 0.06	0.45 ± 0.08	0.30 ± 0.02	0.32 ± 0.01	0.36 ± 0.03	0.37 ± 0.01	0.29 ± 0.02	0.33 ± 0.03	0.32 ± 0.00	0.35 ± 0.01
21	Decanal <n−>	1200.79	**1201**	0.35 ± 0.02	0.33 ± 0.03	0.34 ± 0.01	0.52 ± 0.12	0.35 ± 0.02	0.23 ± 0.02	0.23 ± 0.02	0.24 ± 0.00	0.23 ± 0.03	0.30 ± 0.02	0.26 ± 0.00	0.24 ± 0.00
22	Geraniol	1249.88	**1249**	1.31 ± 0.55	1.33 ± 0.05	1.95 ± 0.67	1.63 ± 0.22	1.68 ± 0.25	1.32 ± 0.04	1.56 ± 0.12	1.87 ± 0.26	**1.25 ± 0.06**	1.40 ± 0.02	**2.15 ± 0.33**	1.55 ± 0.07
23	Camphane	1288.67	**1294**	0.21 ± 0.06	0.28 ± 0.04	0.29 ± 0.03	0.32 ± 0.04	0.29 ± 0.02	0.29 ± 0.02	0.25 ± 0.02	0.192 ± 0.03	0.21 ± 0.00	0.26 ± 0.01	0.26 ± 0.01	0.28 ± 0.01
24	Geranyl acetat	1380.29	**1379**	**3.49 ± 0.37**	4.16 ± 0.03	5.25 ± 0.17	**5.51 ± 0.78**	4.89 ± 0.10	3.90 ± 0.12	5.21 ± 0.32	3.93 ± 0.07	3.59 ± 0.27	3.58 ± 0.02	4.65 ± 0.43	4.82 ± 0.08
25	Tetradecane	1395.09	**1400**	0.59 ± 0.09	0.42 ± 0.00	0.29 ± 0.09	0.38 ± 0.03	0.31 ± 0.11	0.36 ± 0.06	0.41 ± 0.07	0.24 ± 0.07	0.35 ± 0.02	0.34 ± 0.01	0.33 ± 0.01	0.32 ± 0.00
26	*trans*-Caryophyllene	1409.80	**1417**	0.30 ± 0.01	0.31 ± 0.03	0.41 ± 0.10	0.28 ± 0.01	0.29 ± 0.02	0.35 ± 0.02	0.29 ± 0.05	0.30 ± 0.01	0.26 ± 0.00	0.26 ± 0.00	0.27 ± 0.01	0.40 ± 0.10
27	2-Dodecenal	1461.04	**1467**	3.88 ± 0.15	**2.92 ± 0.50**	4.46 ± 0.61	4.20 ± 0.74	3.63 ± 0.13	3.89 ± 0.07	**4.36 ± 0.28**	3.01 ± 0.01	3.69 ± 0.04	3.16 ± 0.16	3.50 ± 0.15	3.42 ± 0.08
	Total indentified compounds (%)			**90.31**	93.62	94.70	90.67	93.60	96.63	92.83	92.78	**97.09**	93.63	95.84	93.06

Significant values are in bold.

*T1* without fertilizer × without HA (control), *T2* without fertilizer × HA before flowering, *T3* without fertilizer × HA at beginning of flowering, *T4* vermicompost × without HA, *T5* vermicompost × HA before flowering, *T6* vermicompost × HA at beginning of flowering, *T7* Livestock manure × without HA, *T8* Livestock manure × HA before flowering, *T9* Livestock manure × HA at beginning of flowering, *T10* chemical fertilizer × without HA, *T11* chemical fertilizer × HA before flowering, *T12* chemical fertilizer × HA at beginning of flowering.

**Table 3:** The macro and micro-nutrient content of coriander plants is influenced by chemical and organic fertilizers and humic acid application (an average over the two years).

Treatments	N (%)	P (%)	K (%)	Fe (mg g^−1^ DM)	Zn (mg g^−1^ DM)	Mn (mg g^−1^ DM)
T1	2.031 e	1.292 g	0.237 de	0.166 g	0.064 e	0.049 e
T2	2.360 d	0.846 h	0.340 d	0.296 f	0.104 d	0.083 bc
T3	2.338 h	1.583 f	0.589 bc	0.601 b	0.159 b	0.116 a
T4	3.332 ab	2.690 ab	0.664 ab	0.528 bc	0.128 c	0.095 b
T5	3.737 a	2.781 a	0.722 a	0.0983 g	0.052 e	0.057 de
T6	2.451 ed	2.13 d	0.340 d	0.299 f	0.094 d	0.083 bc
T7	2.323 h	2.684 ab	0.554 c	0.475 cd	0.129 c	0.095 b
T8	2.560 e	2.571 ab	0.485 c	0.685 a	0.188 a	0.117 a
T9	2.141 g	2.220 d	0.214 e	0.159 g	0.056 e	0.050 e
T10	2.199 ed	2.093 d	0.340 d	0.330 ef	0.104 d	0.073 cd
T11	2.861 c	1.602 f	0.256 de	0.594 b	0.189 a	0.119 a
T12	2.550 d	2.241 cd	0.485c	0.399 de	0.137 c	0.095 b
LSD at 0.05%	0.14	0.107	0.161	0.076	0.0169	0.0159
**Significance**						
Fertilization		**	**	**	**	**
Humic acid		^ns^	^ns^	**	**	^ns^
Fertilization × humic acid		**	**	**	**	**

*T1* without fertilizer × without HA (control), *T2* without fertilizer × HA before flowering, *T3* without fertilizer × HA at beginning of flowering, *T4* vermicompost × without HA, *T5* vermicompost × HA before flowering, *T6* vermicompost × HA at beginning of flowering, *T7* Livestock manure × without HA, *T8* Livestock manure × HA before flowering, *T9* Livestock manure × HA at beginning of flowering, *T10* chemical fertilizer × without HA, *T11* chemical fertilizer × HA before flowering, *T12* chemical fertilizer × HA at beginning of flowering. *, ** and ns, significant at the 5% and 1% probability levels and non-significant, respectively. Different letters indicate significant differences according to LSD test P < 0.05.

**Table 4:** Monoterpenes and sesquiterpenes percentage of coriander essential oil under the influence of organic and chemical fertilizers and humic acid (average of 2 years).

Group of essential oil compounds	Treatments											
	T1	T2	T3	T4	T5	T6	T7	T8	T9	T10	T11	T12
Monoterpene hydrocarbons	15.48	18.38	18.84	18.30	19.45	22.99	22.50	24.21	27.57	25.56	26.82	29.27
Oxygenated monoterpenes	64.89	68.78	67.66	66.13	69.34	69.31	66.26	68.43	70.07	69.99	70.54	66.02
Sesquiterpene hydrocarbons	0.30	0.31	0.41	0.28	0.29	0.35	0.29	0.30	0.26	0.26	0.27	0.40
Other components	9.64	8.15	10.79	10.96	9.52	9.99	10.77	7.84	8.20	7.82	9.21	9.37
Total identified compounds (%)	90.31	93.62	94.70	90.67	93.60	96.63	92.83	92.78	97.09	93.63	95.84	93.06

*T1* without fertilizer × without HA (control), *T2* without fertilizer × HA before flowering, *T3* without fertilizer × HA at the beginning of flowering, *T4* vermicomposting × 0 HA, *T5* vermicomposting × HA before flowering, *T6* vermicomposting × HA at the beginning of flowering, *T7* manure × without HA, *T8* manure × HA before flowering, *T9* manure × HA at the beginning of flowering, *T10* chemical fertilizer × without HA, *T11* chemical fertilizer × HA before flowering, *T12* chemical fertilizer × HA at the beginning of flowering.

### Macro- and micro-nutrients content

Based on the results, the macro-and micro-nutrients contents were significantly affected by the different fertilizers source combined with HA. The highest content of N (3.73%), P (2.78%), and K (0.72%) were observed with the application of vermicompost × HA before flowering. The highest content of Fe (0.685 mg g^−1^ DM), Zn (0.189 mg g^−1^ DM), and Mg (0.119 mg g^−1^ DM) was obtained by using livestock manure × HA before flowering. The lowest content of macro- and micronutrients were noted in the control (Table 3).

### Pearson correlations

The EO constituent results showed positive and significant correlations between coriander GY with EOY and EOC (0.82% and 0.98%, respectively). Linalool content showed remarkable positive correlations with GY, EOC, and EOY (0.68%, 0.89%, and 0.75%, respectively). There were also significant positive correlations between α-pinene content and GY, EOC, and EOY. Similarly, γ-terpinene content showed positive correlations with the GY, EOC, and EOY (Table 5).

**Table 5:** Pearson’s correlation factors (coefficients) between total grain yield, essential oil content, essential oil yield, and chemical composition of coriander essential oil influenced by the chemical and organic fertilizers × humic acid application.

	Grain yield	Essential oil content	Essential oil yield	Linalool	α-Pinene	γ-Terpinene
Grain yield	1					
Essential oil content	0.82**	1				
Essential oil yield	0.98**	0.90**	1			
Linalool	0.68*	0.89**	0.75**	1		
α-Pinene	0.84**	0.71**	0.58*	0.76**	1	
γ-Terpinene	0.75**	0.74**	0.76**	0.64*	0.71**	1

*, **, Significant at 5 and 1% probability levels, respectively. -tailed).

## Discussion

This experiment revealed that organic and chemical fertilizers combined with HA could improve the coriander plant height (Fig. 1a). Studies have shown that the application of organic fertilizer improved soil fertility and microbial flora structure^18^. Vermicompost is an organic fertilizer is a rich source of nutrients, such as N, P, and K, and through the release of some organic acids (e.g., oxalic acid) and by affecting the plant cells metabolism improves the nutrients uptake, photosynthetic activity and metabolic processes of several enzymes and hence influence the plants growth and height^19,20^. Similarly, a study on mint^20^ is consistent with our results. In agreement with our findings, it has been reported that HA can directly improve plant growth by accelerating proteins synthesis, increasing water and nutrient uptake, and enhancing fertilizer use efficiency^21,22^.

In this experiment, lateral branch number was enhanced using different fertilizers source combined with HA (Fig. 1b). Various studies suggested that applying organic fertilizers such as vermicompost and manure improves nutrients availability and root access to the minerals^23^. Thereby, these fertilizers indirectly increase the photosynthetic rate by developing the root system. As a result, more photo-assimilates are stored in the stem, leading to the production of more lateral branch number^24^. In line with our result, a study on coriander indicated that the highest recorded number of lateral stems was obtained by applying organic fertilizers^25^. According to the results, BY and plant DW of the coriander plants increased by using organic and chemical fertilizers × HA, although vermicompost combined with HA improved BY and plant DW more than other treatments (Figs. 1c, 2a). Vermicompost improves the plant quality and yiled by enhancing nutrient availability, mainly Fe and Zn adsorption^26^. Asadi et al.^12^ reported that vermicompost in peppermint significantly increased BY in line with our findings. Additionally, growth stimulants, such as HA induced the biosynthesis of amino acids and ultimately improved BY with the regulation and activation of the proteins' metabolic pathways and enzymes activity^27^. The higher data for lateral branch number in this experiment could be due to the positive role of HA on the metabolism of the plant root system, physiological processes, photosynthetic rate, its hormonal effects, which finally improve BY and plant DW^28^. The same effect of organic fertilizer and HA application was also observed on *Lens culinaris*^29^, *Orthosiphon stamineus* Benth^30^ and *Lycopersicum esculentum*^31^.

The use of chemical and organic fertilizers with high nitrogen content leads to photosynthetic organ development, increasing the production and storage of photo-assimilates^32^. Our results revealed that TSW positively correlated with the GY (Table 5). N availability is essential for flowering and pollination and even transferring assimilates and filling seeds. So, nitrogen-containing fertilizers play a crucial role in the number and yield of seeds. Moreover, the TSW was correlated to the nitrogen available during the growth stage^33^. Consistent with the current experiment, it has been reported that HA increased the TSW in canola^34^. Moreover, vermicompost can also increase N, P, and K content in the soil and promotes plant growth and yield^35^.

Chemical and organic fertilizers boost the content of the photosynthetic pigment by enhancing nitrogen absorption, increasing the light acquisition, assimilating production, and improving growth and yield^36^. Furthermore, the high photosynthesis potential under organic fertilizers is probably due to the stimulated activity of beneficial soil microorganisms, enhancing chlorophyll content in the plants^37^. It was found that photosynthesis pigments content was increased by using the organic and chemical fertilizers in combination with HA (Fig. 3a–c). Similarly, it has been stated that the application of vermicompost significantly increased the contents of chlorophylls and carotenoids in *Borago officinalis*^38^ and *Lactuca sativa*^39,40^. HA facilitates the transfer of nutrients through the chelation and reduces evapotranspiration^41^. Ali et al.^41^ in sorghum and Mahmood et al.^42^ in corn also observed that HA application increased the content of chlorophylls.

Several studies have pointed out the role of chemical and organic fertilizers in increasing the protein content of plants. It has been reported that vermicompost increases the concentrations of N and K in plants^43^. The adequate N content in the soil resulted in the soluble protein content, and also TSP content was improved with enhancing N per unit area of ​​the leaf^44^. K also plays an essential role in plant metabolism and is one of the critical components in protein synthesis^45^. The results indicated that vermicompost and HA could enhance the TSP in coriander plants (Fig. 4a). HA enhances protein synthesis through a number of biochemical mechanisms, such as the adsorption of active ions^46^. Therefore, there may be another reason for increasing TSP in our study. Similar to our results, an enhancement of TSP content has been reported with vermicompost and HA in Chinese cabbage^47^, peanuts^22,48^, and sorghum^49^.

According to the results, the co-application of organic fertilizers and HA positively influenced coriander GY and EOY (Figs. 2c, 4d). Organic fertilizers provide moisture, prepare the soil substrate for better root and shoot growth and improve the yield due to the supply of nutrients, such as N^50^. Fertilizers such as nitrogen are essential for the production of structural proteins, and also are needed for the overall growth, development and yield of plants^51^. In addition, vermicompost increases the grain growth and yield by improving the availability of certain nutrients, in particular, Fe and Zn, and in turn by a direct effect on the plant metabolism. The application of HA increased nutrient uptake, photosynthesis pigments content, plant growth, and GY (Fig. 2c). HA probably affects the activity of Rubisco enzyme, which positively influences the photosynthetic potential, absorption of macro-and micronutrients, activity of some enzymes, cell membrane permeability, and eventually improves GY^20^.

Essential oils belong to the terpenoids, a significant class of plant secondary metabolites. The synthesis of terpenes precursors i.e., isopentenyl pyrophosphate (IPP) and dimethylallyl pyrophosphate (DMAPP) need ATP and NADPH as photosynthesis product. Thus, photosynthesis potential directly affects the production of EO. Moreover, CO_2_ and glucose are the initial precursors in forming essential oils^52^. The different fertilizer sources and HA applications increased the EOC and EO components of *Ocimum basilicum* var. purple^53^, *Arachis hypogaea* L.^54^, and *Salvia officinalis*^13^, which agree with the present research (Fig. 4b and Table 2). The use of organic fertilizers such as vermicompost and HA can also increase the EOC by enhancing the uptake of P and N, which are the major prerequisites for the primary and secondary metabolism in most medicinal plants^13,55^.

In addition, the highest concentration of micro-elements (Fe, Zn, and Mn) was obtained by applying vermicompost × HA before flowering (Table 3). The different fertilizer sources improved micro-elements content due to enhancing the cation exchange capacity of the soil, the gradual release of nutrients, and the biological activities and physicochemical properties of the soil^56^. According to these results, an increase in the macro-elements (e.g., N, P, and K) has also been reported in peppermint plants treated with organic and chemical fertilizers^57^. The results also showed that HA foliar application significantly affected the content of macro-and microelements compared to the control.

In conclusion, our study focused on the effect of the fertilizer source × HA on the nutrients absorption, growth responses, and the content, yield, and composition of coriander essential oil. It van be concluded from the present study that the application of vermicompost × HA fertigation before flowering improved the morphological trait, total soluble protein content, photosynthesis pigments and macro- and micro-nutrient contents. Furthermore, the chemical fertilizer and vermicompost × HA fertigation at the beginning of flowering increased the content and yield of coriander essential oil. The main idea of sustainable agricultural systems is to reduce the use of chemical fertilizers. The current experiment exhibited that chemical fertilizers could be replaced by vermicompost, livestock manure, and HA. The overall results could be advisable to the extension section, and the pioneer farmers to reduce the chemical fertilizers input and secure environmental health. However, many new studies are required for the evaluation of the efficiency of humic acid application and other organic fertilizer sources at different growth stages can be realized on a large scale.

## Materials and methods

### Study site and treatment

The experiment was conducted during the two successive seasons of 2018 and 2019 in the research farm of the University of Maragheh, East Azerbaijan Province, Iran (E 46° 16′ E; N 37° 23′, 1485 m above sea level.). The soil Physico-chemical characteristics of the experimental site are listed in Table 6. The soil was composed of sandy clay loam with pH 8.16, 1.23% organic carbon, 0.09% total N, 11.05 and 570.85 mg/kg of available P and K, respectively (depth of 0–30 cm). The climatic data in the research area are presented in Table 7.

**Table 6:** Monthly average temperature and total monthly precipitation of experimental site during 2018–2019.

Year	April	May	June	July	August	September	October
**Monthly average temperature (°C)**							
2018	12.6 $\pm$ 5.2	16.6 $\pm$ 6.3	24.1 $\pm$ 7	30.2 $\pm$ 7.05	27.7 $\pm$ 7	23.6 $\pm$ 6.95	15.9 $\pm$ 5.9
2019	10.4 $\pm$ 4.5	18.5 $\pm$ 5	25.7 $\pm$ 5.1	27.6 $\pm$ 5.3	27.8 $\pm$ 6	22.1 $\pm$ 4.9	16.7 $\pm$ 4.5
10-year mean	12.7 $\pm$ 5.1	17.9 $\pm$ 5.4	23.9 $\pm$ 5.9	27.9 $\pm$ 6.3	27.4 $\pm$ 6.2	22.0 $\pm$ 5.7	15.0 $\pm$ 5.1
**Total monthly precipitation (mm)**							
2018	44.9	54.5	1.7	0.1	0	0.2	19.5
2019	51.3	37.8	4.2	0.0	0.0	0.0	6.3
10-year mean	39.4	16.6	3.4	0.4	0.3	1.7	17.1

**Table 7:** The physicochemical properties of the field soil used in this experiment.

Depth soil (cm)	EC (dS/m)	pH	Organic carbon (%)	N (%)	P (%)	K (mg kg^−1^)	Soil pattern
0–30	1.36	7.69	0.72	0.07	20.18	805.6	Lumi

This research was conducted as a factorial experiment based on a randomized complete block design (RCBD) with three replications. Experimental factors included fertilizer type and HA including: (T1) without fertilizers × without HA (control), (T2) without fertilizers × HA before flowering (200 mg L^−1^), (T3) without fertilizers × HA at the beginning of flowering (200 mg L^−1^), (T4) vermicompost (1.5 kg m^−2^) × without HA, (T5) vermicompost × HA before flowering (T6) vermicompost × HA at the beginning of flowering, (T7) manure (4 kg m^−2^ of livestock manure) × without HA, (T8) manure × HA before flowering (T9), manure × HA at the beginning of flowering (T10) chemical fertilizers (20 g m^−2^ of urea and 10 g m^−2^ of triple superphosphate) × without HA, (T11) chemical fertilizers × HA before flowering and (T12) chemical fertilizers × HA at the beginning of flowering. The humic acid fertigation was applied before flowering 60 days after sowing and was used 70 days after sowing for the beginning of flowering. The chemical fertilizers were applied according to the chemical and physical soil analysis. Humic acid was prepared from Humic Miracle, whose chemical characteristics are presented in Table 8. The livestock manure, vermicompost, and chemical fertilizers were applied during farm preparation.

**Table 8:** The chemical properties of fertilizers used in the present experiment.

Humic acid (%)	Folic acid (%)	Organic matter (%)	Potassium oxide (%)
70	2%	1.7	12

The landrace coriander seeds were collected from East Azerbaijan Province. In May of 2018 and 2019, 36 experimental plots were prepared. Vermicompost and livestock manure were added one month before planting and triple superphosphate at the planting time, but urea was applied 20 and 40 days after sowing as top-dressing. Each plot (2 × 3 m^2^) consisted of 7 rows with 35 cm row distance and 15 cm within rows. After planting the seeds, the plots were irrigated by the drip irrigation method. Operations such as irrigation, weed control, etc. were performed regularly during the growing season. All agronomic practices were performed uniformly for all plots.

### Measurement of growth parameters

After full maturity (110–120 days after sowing), all experimental plants were separately harvested, and then yield, and its components were recorded. Traits such as plant height, stem diameter, shoot dry matter (DM), biological yield (BY), thousand-seeds weight (TSW), and grain yield (GY) were recorded on five plants per plot at the physiological maturity and harvest time.

### Photosynthetic pigments content

Chlorophylls (Chl *a* and *b*) and carotenoids (CARs) content were determined spectrophotometrically using equations described by the Arnon method^58^. Leaf sample (0.5 g) was ground by liquid nitrogen and was suspended in 10 ml of %80 acetone. Their content was determined by measuring the extinction of the extract at the significant red absorption maxima of Chl *a* (664 nm) and *b* (647 nm) and CARs (470) and inserting these values simultaneously into the following Eqs. (1, 2 and 3).

1 $$\text{Chlorophyll} \; a \; \text{mg}/\text{kg} \; \text{FW} = [12.7({\text{A}}663) - 2.69({\text{A}}645)],$$

2 $$ {\text{Chlorophyll}}\,{\text{b}}\,{\text{mg}}/{\text{kg}}\,{\text{FW }} = \, \left[ {21.50 \, \left( {{\text{A}}_{{{645}}} } \right) \, - 5.10\left( {{\text{A}}_{{{663}}} } \right)} \right], $$

3 $$\text{Carotenoids}= [1000({\text{A}}470) -1.82\text{Ca} - 85.02\text{Cb}] /198$$

### Total soluble proteins (TSP) content

Fresh leaf samples (0.2 g) were grounded by liquid nitrogen and homogenized in 1.5 ml 50 mM Na buffer phosphate (pH:7.8) including 1 mM EDTA and 2% (w/v) polyvinylpolypyrolidone. The homogenate was centrifuged at 12,000 rpm for 15 min at 4 °C. Supernatants were employed for TSP content based on the Bradford method^59^. The absorbance was read at 595 nm and expressed as mg g^−1^ FW. Bovine serum albumin (BSA) was used as standard so that, six standard solutions containing 0, 0.2, 0.4, 0.6, 0.8 and 1 mg ml^−1^ prepared. 100 μl Bradford solution was added to each of the standards..

### Essential oil distillation and analysis

The harvested seeds (40 g) were hydro-distilled to determine the percentage of essential oil using the Clevenger of British Pharmacopoeia for 3 h. Anhydrous sodium sulfate was added to each distillated essential oil and then stored at 4 °C before analysis to remove the water droplets. The essential oil content and yield were calculated using the following formulas:

$$ {\text{Essential}}\,{\text{oil}}\,{\text{content }}\left( \% \right) \, = \, \left( {{\text{distillated}}\,{\text{essential}}\,{\text{oil }}\left( {\text{g}} \right)/{4}0\,{\text{g}}} \right) \, \times { 1}00, $$

$$ {\text{Essential}}\,{\text{oil}}\,{\text{yield }}\left( {{\text{g}}\,{\text{m}}^{ - 2} } \right) = \frac{{{\text{mass}}\,{\text{of}}\,{\text{distillated}}\,{\text{essential}}\,{\text{oil }}\left( {\text{g}} \right)}}{{{\text{mass}}\,{\text{of}}\,{\text{grain}}\,{\text{yield}}\left( {\text{g}} \right)}} \times 100. $$

### GC–MS analysis

The essential oils were analyzed using GC–FID and GC–MS. The analysis was conducted using an Agilent 7990 B gas chromatograph equipped with a 5988A mass spectrometer and a HP-5MS (0.25 mm i.d., 30 mL, 0.25 μm f.t., 5% phenyl methyl polysiloxane). The following oven temperature was used: 5 min at 60 °C, then up to 240 °C with the rate of 3 °C min^−1^, held for 10 min. Helium (carrier gas) flow rate was 1 mL min^−1^; the injector split ratio was 1:30; the mass range and electron impact (EI) were 400 *m/z* and 70 eV, respectively. The identification of constituents was performed using the procedure explained by Morshedloo et al.^60^, which is based on the interactive combination of linear retention indices (RIs), calculated respect to a homologous series of n-alkanes (Supelco, Bellefonte, CA), and the mass spectrum (MS) matching with commercial libraries (ADAMS, WILEY 275 and NIST 17). GC-FID analysis was performed using an Agilent 7990 B gas chromatograph equipped with a flame ionization detector (FID), capillary column VF 5MS (30 mL, 0.25 mm i.d., 0.50 μm f.t., 5% phenyl methyl polysiloxane). The same oven temperature reported for GC–MS was used. The injection volume of the essential oil was 1 μL the essential oil in *n*-hexane (1:100). Quantification of the constituents was performed by peak area normalization without using correction factors^61^.

### Statistical analysis

All data were subjected to a normality test via the Anderson–Darling method, and homogeneity of data was checked through Levene’s test. Then, data were subjected to combined ANOVA using MSTAT-C Software. The significant differences among means were compared with the Last Significant Difference (LSD) test at P < 0.05. Pearson's correlation coefficient was calculated between grain yield, essential oil content, essential oil yield, and significant constituents of coriander essential oil. Since the effect of time (year) in the combined analysis of the experiment was not significant, the average data of two years were analyzed as a factorial experiment based on a randomized complete block design (RCBD).
